# Trajectories of job demands and control: risk for subsequent symptoms of major depression in the nationally representative Swedish Longitudinal Occupational Survey of Health (SLOSH)

**DOI:** 10.1007/s00420-017-1277-0

**Published:** 2017-11-11

**Authors:** Julia K. Åhlin, Hugo Westerlund, Yannick Griep, Linda L. Magnusson Hanson

**Affiliations:** 1 Stress Research Institute, Stockholm University, SE-106 91 Stockholm, Sweden; 2 Department of Psychology, University of Calgary, 2500 University Drive NW, Calgary, AB T2N 1N4 Canada

**Keywords:** Depressive symptoms, Demand-control model, Job strain, Work stress, Longitudinal studies, Latent class growth analysis

## Abstract

**Purpose:**

Depression is a global health concern. High job demands, low job control, and the combination (high strain) are associated with depression. However, few longitudinal studies have investigated changed or repeated exposure to demands and control related to depression. We investigated how trajectories of exposure to job demands and control jointly influence subsequent depression.

**Methods:**

We included 7949 subjects from the Swedish Longitudinal Occupational Survey of Health, who completed questionnaires of perceived job demands and control, and depressive symptoms from 2006 to 2014. None of them were depressed between 2006 and 2012. Univariate and joint group-based trajectory models identified groups with similar development of demands and control across 2006–2012. Logistic regression estimated the risk for symptoms of major depression in 2014 according to joint trajectory groups.

**Results:**

The joint trajectory model included seven groups, all with fairly stable levels of demands and control over time. Subjects in the high strain and active (high demands and high control) trajectories were significantly more likely to have subsequent major depressive symptoms compared to those having low strain, controlling for demographic covariates (OR 2.15; 95% Cl 1.24–3.74 and OR 2.04; 95% CI 1.23–3.40, respectively). The associations did not remain statistically significant after adjusting for previous depressive symptoms in addition to demographic covariates.

**Conclusions:**

The results indicate that the levels of job demands and control were relatively unchanged across 6 years and suggest that long-term exposure to a high strain or active job may be associated with increased risk for subsequent depression.

**Electronic supplementary material:**

The online version of this article (doi:10.1007/s00420-017-1277-0) contains supplementary material, which is available to authorized users.

## Introduction

Depression is a common, disabling and burdensome mental disorder (Wittchen et al. 2011), and thus a major public health concern.

Increased severity of depression has been associated with more disability, unemployment and poorer work performance (Birnbaum et al. 2010). Occupational stress research has shown that both acute work-related stressful experiences and enduring structural occupational factors can contribute to depression (Tennant 2001). The majority of studies draw upon the Demand-Control Model (Karasek 1979) encompassing the psychological demands and control dimensions. Psychological job demands refer to the pace and mental intensity of work, whereas job control (decision latitude) comprises decision authority and skill discretion. The model classifies jobs into four categories: “high-strain,” “low strain,” “active,” and, “passive” jobs (Karasek 1979). “High-strain” jobs are characterised by high demands and low control, whereas “low strain” reflect low demands and high control (Karasek 1979). “Active” jobs involve high demands and high control, whereas “passive” involve low demands and low control (Karasek and Theorell 1990). According to the model, especially “high strain” has been suspected to cause mental strain, and if prolonged, constitute a health risk (Karasek and Theorell 1990). In contrast, “low strain” jobs may be associated with lower health risks (Karasek and Theorell 1990).

Previous research indicates that high demands, low control, and “high strain” are risk factors for depressive symptoms (Bonde 2008; Netterstrom et al. 2008; Nieuwenhuijsen et al. 2010; Stansfeld and Candy 2006; Theorell et al. 2015). A recent review found that job strain and low decision latitude influenced the development of depressive symptoms, while evidence for a negative influence of psychological demands was limited (Theorell et al. 2015). In another review, the exposure to high demands was a stronger predictor of depression than the exposure to low control (Netterstrom et al. 2008). However, very few longitudinal studies have investigated how changed or repeated exposure to job strain over time is associated with subsequent depression. Although some studies suggest that accumulated or increased job strain is associated with depression, results have been inconsistent and few included more than two measurement points (Burns et al. 2016; Stansfeld et al. 2012), hampering the ability to properly examine accumulation or change over time. Therefore, we know little about how the duration of, and change in demands and control relate to the risk for developing depression (Netterstrom et al. 2008; Nieuwenhuijsen et al. 2010; Wang et al. 2009). The aim of the present study was to increase the knowledge about the role of demand/control dynamics by investigating how trajectories of job demands and control jointly influence the risk for subsequent depression.

## Materials and methods

### Study population

We used data from the Swedish Longitudinal Occupational Survey of Health (SLOSH) cohort, a longitudinal survey of working life and health initiated in 2006 (Magnusson Hanson et al. 2008). SLOSH consists of participants in the Swedish Work Environment Surveys (2003–2011), originally representative of the Swedish working population. Participants are followed up every other year and depending on their current situation at follow-up, respondents chose between two versions of the questionnaire: (1) ‘in paid work’ (i.e. gainful employment for at least 30% of full-time), or (2) ‘not in paid work’ (i.e. currently not working or working less than 30% of full-time). Five waves of data have been used in the present study: 2006, 2008, 2010, 2012, and 2014 (number of invited participants = 9154, 18639, 20298, 17434 and 38659, respectively). The overall response rates were between 65 and 57%, resulting in a sample of 7949 SLOSH participants who: (1) responded to the ‘gainfully employed’ questionnaire during any of the first four waves (2006–2012) (to model their demand and control trajectories during this time period), (2) responded to either of the questionnaires in the fifth wave (2014), and (3) did not reach a symptom score indicating major depression in any of the first four waves (exclusion of 777 participants) (See Online resource 1). The reason that we did not require participants to have responded in all four waves was because the analytical strategy (described in the Statistical methods section) can handle missing data. A larger proportion of the excluded individuals compared to the 7949 included were young (14.7%) and middle-aged (41.7%), women (72.1%), single/living alone (27.9%) and had children (51.3%). Also, high demands (62.8%) and low control (63.3%) were more common and depressive symptoms were significantly higher (mean = 14.3, Sd = 6.3) among the excluded (*p* < 0.05 based on *χ* ^2^ tests and *t* test). The study was approved by the Regional Ethical Review Board in Stockholm, dnr: 2006/158-31, 2008/240-32, 2010/0145-32, 2012/373-31/5, 2013/2173-32, 2015/2187-32.

### Main measures

Job demands and job control were measured using the Swedish shortened version of the Demand-Control-Support-Questionnaire (Chungkham et al. 2013; Fransson et al. 2012; Sanne et al. 2005) in wave one through four. Job demands were measured by four items (e.g., Do you have to work very intensively?). Job control was measured by six items (e.g., Do you have a choice in deciding how you do your work?) Items were scored on a Likert scale ranging from (1) “never/almost never” to (4) “often”. The scales showed acceptable alpha coefficients, which were mean *α* = 0.67, Sd = 0.02, range 0.65–0.69 for demands and mean α = 0.66, Sd = 0.00, range 0.65–0.66 for control. The level-specific omega reliability for job demands (*ω* = 0.89) and job control (*ω* = 0.95) was also satisfactory, indicating satisfactory reliability when also accounting for the multilevel nature of the data (Geldhof et al. 2014).

Symptoms of depression were measured using the SCL-Core Depression scale (SCL-CD), a brief 6-item subscale of the (Hopkins) Symptom Checklist (SCL) depression scale (Magnusson Hanson et al. 2009, 2014). Only in wave 5, respondents were instructed to indicate to what extent they were feeling blue, feeling no interests in things, feeling lethargy or low in energy, worrying too much about things, blaming oneself for things, and feeling everything is an effort (*α* = 0.89). Items were scored on a Likert scale ranging from (1) “not at all” to (4) “extremely”. We used a sum scale assessing severity of depression and a cut-off score of ≥ 17 to indicate symptoms of major depression, in line with a previous study examining the most suitable threshold value for major depression in epidemiological research (Magnusson Hanson et al. 2014).

### Statistical methods

To investigate how levels of job demands and control changed over time, group-based trajectory modelling (GBTM) was used. GBTM was developed to study a behaviour/phenomenon which is repeatedly measured over time, and identifies subgroups of individuals following a similar developmental course over time or age (Nagin 2005). We conducted GBTM using the STATA TRAJ plugin (Jones and Nagin 2012). First, we fit univariate trajectory models for demands and control over calendar time, respectively. The identified univariate trajectory models were subsequently used to estimate a joint trajectory model, in which we simultaneously assessed the trajectories of demands and control. Finally, we investigated the association between the joint trajectories and symptoms of major depression using multiple logistic regression analysis.

#### Univariate trajectory analysis

To determine the optimal number of trajectory groups, and to test the level of complexity (i.e., the number and order of regression parameters) required to describe the demand and control trajectories, we followed a similar procedure as previously described in the literature (Andruff et al. 2009; Nagin 2005). We applied a censored normal distribution because demands and control were assessed by composite psychometric scales (Nagin 1999). First, we fit a single trajectory model for demands and control, respectively. Because we used four waves of data, we initially tested a cubic polynomial shape (third-order polynomial), followed by a quadratic, and linear shape. When the cubic parameter was significant, the same (single trajectory) model was compared to a two-trajectory model. We repeated this procedure until there was no longer evidence for improvement in fit. However, if cubic component(s) were not significant, we tested a quadratic trajectory. The same procedure would then be repeated for non-significant quadratic components (i.e., replaced by linear). Only when all components in each model were significant at *p* < 0.05, we compared that model to a model with one additional trajectory group (Nagin 2005). Linear components were always retained irrespective of statistical significance (Louvet et al. 2009).

We chose the Bayesian Information Criterion (BIC) as a fit index for determining the best model fit (Raftery 1995; Schwarz 1978). More specifically, we used an estimate of the log Bayes factor (2log_e_(B_10_) ≈ 2(ΔBIC) (Kass and Raftery 1995; Raftery 1995). This estimate approximately equals 2(BIC_complex model_–BIC_null model_) (Andruff et al. 2009; Jones et al. 2001). We then interpreted the log Bayes Factor estimates (values 2–6 reflect positive evidence, 6–10 reflect strong evidence, and > 10, reflect very strong evidence against the null model) as the degree of evidence favouring the more complex model, ensuring model parsimony (Jones et al. 2001). However, because BIC sometimes keeps improving when adding trajectory groups (Nagin 2005), we stopped adding groups when the model no longer captured new distinctive features of the data, when some trajectory group became smaller than 1%, when entropy (i.e., index of classification accuracy ranging from 0 to 1 with values closer to 1 indicating better precision; (Jung and Wickrama 2008) or average posterior probabilities of assignment (APPA; preferably > 0.7; (Nagin 2005) deteriorated. Hence, several different statistical criteria were used to identify the best trajectory models, combined with an assessment of whether the data distinguished distinctive features in parsimonious way.

#### Joint trajectory analysis

After selecting the univariate trajectory models, we fit joint trajectory models to investigate the linkages between trajectories of job demands and control. The number of joint trajectory groups that were tested was limited based on the identified univariate trajectory groups since it has been shown that joint trajectories and the corresponding univariate trajectories do not differ much (Nagin and Tremblay 2001). In line with our research question, we used a constrained joint model in which each trajectory for demands is uniquely associated with a trajectory for control (Brame et al. 2001; Jones and Nagin 2007; Nagin 2005).

#### Association between joint trajectories and depression

To investigate the relationship between the joint trajectory groups and subsequent symptoms of major depression (as indicated by a symptom score ≥ 17), we conducted multiple logistic regression analysis. Trajectory groups served as the predictor variable and symptoms of major depression in wave five as the outcome variable. First, we fit a crude model. Second, we ran a model adjusting for previous level of depressive symptoms, to reduce the risk that depressive symptoms affected the ratings of job demands and control (Rugulies et al. 2006). In the third model, we included the demographic covariates age, sex, having children at home (“Do you have any children living at home? Include children living with you at least half of the time”), and civil status (“Are you single or married/cohabiting?”) because those kinds of factors are potential confounders according to previous work (Allen et al. 2000; Bonde 2008) and were significantly associated with both perceptions of demands and control, as well as symptoms of major depression. Socioeconomic index was not included as a covariate since it was not associated with perceptions of demands and control, or depression. Fourth, we adjusted for previous depressive symptoms in addition to the demographic covariates. We included covariates, as well as previous depressive symptoms from the second wave, in which the cohort was boosted with new subjects, or in case data were missing in the second wave, we used the earliest wave with available data.

## Results

### Sample characteristics

Among the 7949 included participants, 160 (2%) had (incident) symptoms of major depression in wave 5 [116 (1.5%) had missing data]. Table 1 presents the characteristics of the study sample in wave two (when the cohort was boosted with new subjects), and stratified by symptoms of major depression in wave five. Women were significantly more depressed than men. The distribution of symptoms of major depression was significantly different between the age groups. The highest proportion of having symptoms of major depression was found among individuals younger than 35 years, whereas the lowest was found in those older than 50 years. Individuals with children at home had a significantly higher prevalence of symptoms of major depression than those without children. Those perceiving high job demands in wave two had significantly higher prevalence of symptoms of major depression than those with low demands.

**Table 1:** Characteristics of the study sample in wave two (*N* = 6371), stratified by symptoms of major depression status in wave five

	Total		Major depression in wave 5		No major depression in wave 5		Test of difference
	*N*	%	*N*	%^a^	*N*	%^a^	*p* value
Total subjects in wave 2^b^	6371	–	121	1.9	6164	98.1	–
Sex^b^							
Men	2797	43.9	38	1.4	2725	98.6	0.005
Women	3574	56.1	83	2.4	3439	97.6	
Age group^b^							
< 35 years	640	10.0	21	3.3	608	96.7	0.002
35–49 years	2150	33.8	49	2.3	2089	97.7	
≥ 50 years	3581	56.2	51	1.4	3467	98.6	
Marital status^b^							
Single	1238	19.6	31	2.5	1187	97.5	0.090
Married/cohabiting	5069	80.4	90	1.8	4916	98.2	
Children^b^							
Children at home	2813	44.7	69	2.5	2725	97.5	0.003
No children at home	3486	55.3	49	1.4	3374	98.6	
Socio-economic group^b^							
Unskilled employees	906	14.7	20	2.3	863	97.7	0.963
Skilled employees	917	14.9	19	2.1	886	97.9	
Assistant non-manual employees	732	11.9	15	2.1	716	97.9	
Intermediate non-manual employees	1933	31.5	35	1.8	1876	98.2	
Professionals/upper-level executives	1265	20.6	22	1.8	1229	98.2	
Self-employed	393	6.4	8	2.1	380	97.9	
Job demands and control^b^							
High demands	2593	44.7	59	2.3	2506	97.7	0.048
Low demands	3202	55.3	50	1.6	3113	98.4	
Low control	3227	55.6	58	1.8	3129	98.2	0.477
High control	2575	44.4	53	2.1	2495	97.9	

	Mean	Sd	Mean	Sd	Mean	Sd	
Depressive symptoms	4.57	4.10	8.82	4.17	4.48	4.06	0.000

^a^ The percentages in the fourth and sixth columns refer to the proportion of individuals with and without symptoms of major depression in wave five, in each category presented

^b^ Missing information: missing depression status in wave 5 among the 6371 participants included in wave 2 (*n* = 86, 1.3%). Missing depression status in wave 5 according to wave 2 sex (*n* = 86, 1.3%), age groups (*n* = 86, 1.3%), marital status (*n* = 83, 1.3%), children (*n* = 82, 1.3%), socio-economic group (*n* = 77, 1.3%), job demands (*n* = 67, 1.2%) and job control (*n* = 67, 1.2%)

### Univariate trajectory models of job demands and control

Although BIC kept improving when adding additional groups, the optimal job demands model was considered the one that consisted of six groups (shown in Online Resource 2). In the seven-group model, there were no new distinct features present, and the entropy and APPA decreased. The six trajectories of demands (APPA = 0.71, entropy = 0.63) are shown in Online Resource 3. With the exception of two decreasing trajectories, the cubic, quadratic, and linear terms were often significant for all groups in addition to the intercept, but the visual inspection of the trajectories indicated that the job demands remained approximately at the same level across all waves.

The optimal job control model also consisted of six groups (shown in Online Resource 2), although BIC kept improving when adding groups. In the seven-group model, two groups only included 1–1.1% of the sample, which was considered too small (Jung and Wickrama 2008). In addition, APPA values (mean = 0.77) and entropy (0.67) were somewhat larger in the six-group model. The six control trajectories are shown in (shown in Online Resource 4). The graph illustrates one slightly decreasing, and one increasing group between wave one and two. For the other groups, cubic/quadratic/linear terms were often significant in addition to the intercept, but the estimations were very small in nature and the overall trajectories thus appeared to be stable across time.

### Joint trajectory model

**Fig. 1:**
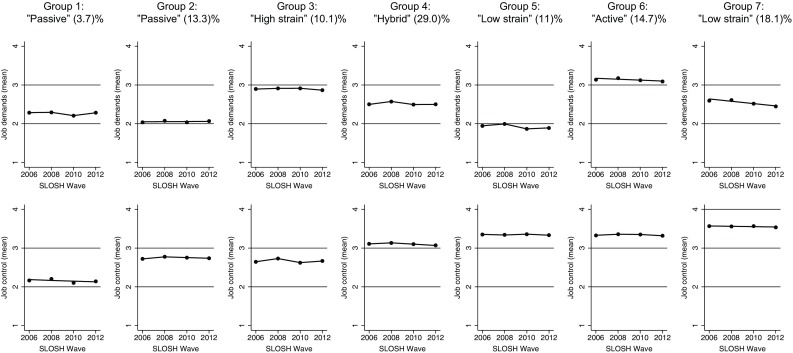
Joint trajectory model of job demands and control in the SLOSH study. Mean level of job demands and control across 6 years according to joint trajectory group, trajectory labels, and the proportion of individuals in each group

Based on the same criteria as described above, the optimal joint trajectory model included seven groups (shown in Online Resource 2). Because BIC improved with the addition of groups, we scrutinized the APPAs and entropy values. The seven-group model had the highest entropy (0.67), relatively large group sizes, and mean APPA = 0.77. The first and second groups were found stable as indicated by only the intercepts being significant, and the remaining five trajectories had significant linear/quadratic/cubic terms. However, the estimations of these higher-order polynomials were very small, resulting in trajectories that have fairly stable levels over time. The joint trajectory groups are presented in Fig. 1. We labelled the joint trajectories based on the demand-control model and according to the quadrant approach, which divides demands and control at the median because this is the most common operationalisation (Courvoisier and Perneger 2010). The median for job demands across wave one through four was 2.50 and for control 3.17. The first group (*N* = 294, 3.7%) thus represented individuals with “passive” jobs because this group had low demands and (very) low control (mean control = 2.13). The second group (*N* = 1057, 13.3%) was also a passive group but with slightly higher level of control (mean control = 2.72). The third group (*N* = 803, 10.1%) represented individuals with high strain jobs because it had high demands and low control. The fourth group (*N* = 2305, 29.0%) represented a hybrid group because both demand and control levels were close to the median. The fifth group (*N* = 874, 11.0%) represented individuals with “low strain” jobs because this group had low demands (mean demands = 1.87) and high control. The sixth group (*N* = 1169, 14.7%) represented individuals with “active” jobs because individuals had high demands and (quite) high control. The last group (*N* = 1439, 18.1%) represented another low strain group although demands were close to the median (mean demands = 2.53).

### Characteristics of the joint trajectories

Table 2 depicts characteristics of the joint trajectory groups. *χ* ^2^ tests showed significant differences between the trajectory groups in terms of distribution of sex, age groups, civil status, and socio-economic index. Before conducting logistic regression, we merged group five and seven to form a larger reference group because they both represented “low strain”. Although we also found multiple passive groups, we did not merge them because they were characterised by more pronounced differences in terms of demands and control.

**Table 2:** Characteristics of the seven joint trajectory groups in wave two

	Joint trajectory groups						
	Passive (1)	Passive (2)	High strain (3)	Hybrid (4)	Low strain (5)	Active (6)	Low strain (7)
	%	%	%	%	%	%	%
Sex							
Men	46.2	40.9	37.1	44.8	45.4	41.4	52.0
Women	53.8	59.1	62.9	55.2	54.6	58.6	48.0
Age group							
< 35 years	13.1	9.8	10.1	7.2	6.9	7.8	7.0
35–49 years	27.3	26.0	25.0	26.1	24.6	32.1	27.9
≥ 50 years	59.6	64.2	64.9	66.7	68.5	60.0	65.1
Civil status							
Single	32.0	23.3	24.0	19.8	19.5	15.3	15.2
Married/cohabiting	68.0	76.7	76.0	80.2	80.5	84.7	84.8
Children							
Children at home	39.7	39.5	41.5	43.7	41.4	53.3	47.8
No children at home	60.3	60.5	58.5	56.3	58.6	46.7	52.2
Socio-economic group							
Unskilled employees	53.6	28.7	28.5	12.8	10.6	5.0	2.7
Skilled employees	12.3	17.8	22.2	18.7	13.0	9.8	8.3
Assistant non-manual employees	20.0	23.5	15.3	12.3	10.9	5.1	5.0
Intermediate non-manual employees	11.8	19.9	25.7	33.7	35.6	40.0	34.3
Professionals/upper-level executives	1.4	6.3	7.6	17.3	20.1	34.0	37.3
Self-employed	0.9	3.8	0.7	5.3	9.8	6.2	12.4

### Joint trajectories and the association with subsequent symptoms of major depression

Table 3 presents the logistic regression models with odds ratios (OR) and 95% confidence intervals (CI). In the unadjusted model 1, subjects belonging to the high strain (OR 2.47; 95% CI 1.45–4.24) and active trajectories (OR 2.18; 95% CI 1.32–3.61) were significantly more likely to have symptoms of major depression in wave five, compared to the reference group (low strain). However, when adjusting for previous depressive symptoms, (model 2) the associations were no longer statistically significant although the OR was largest for high strain (OR 1.35; 95% CI 0.78–2.37). When demographic covariates (age, sex, children and civil status) were included but not previous depressive symptoms (model 3), the high strain (OR 2.15; 95% CI 1.24–3.74) and active trajectories (OR 2.04; 95% CI 1.23–3.40) were still significant predictors. Finally, when adjusting for previous depressive symptoms and demographic covariates (model 4), the associations were no longer statistically significant. However, the ORs for the high strain (OR 1.27; 95% CI 0.72–2.24) and active trajectories (OR 1.29; 95% CI 0.76–2.19) indicated a slightly increased risk of later depression.

**Table 3:** Results from logistic regression analysis predicting major depression in wave five presented as odds ratios (OR) and 95% confidence intervals (CI)

Joint trajectory groups	Depression cases *n* (%)	Modell 1		Modell 2		Modell 3			Modell 4	
		OR	CI	OR	CI	OR		CI	OR	CI
Low strain (5 and 7) reference	30 (1.4)									
Passive (1)	7 (2.6)	1.91	0.83–4.39	1.27	0.54–2.97	1.74		0.75–4.04	1.22	0.52-2.90
Passive (2)	16 (1.6)	1.18	0.64–2.17	1.09	0.58–2.03	1.14		0.61–2.12	1.07	0.57-2.01
High strain (3)	25 (3.3)	**2.47**	1.45–4.24	1.35	0.78–2.37	**2.15**		1.24–3.74	1.27	0.72-2.24
Hybrid passive/high strain (4)	50 (2.0)	1.47	0.93–2.32	1.12	0.70–1.80	1.43		0.90–2.27	1.13	0.70-1.83
Active (6)	32 (2.9)	**2.18**	1.32–3.61	1.27	0.76–2.14	**2.04**		1.23–3.40	1.29	0.76-2.19
Covariates^a^										
Previous depressive symptoms		–	–	**1.24**	1.19–1.28		–	–	**1.23**	1.18–1.27
Age (continuous)		–	–	–	–		**0.97**	0.95–0.98	**0.97**	0.96–0.99
Women		–	–	–	–		**1.57**	1.12–2.20	1.38	0.97–1.95
No children at home		–	–	–	–		0.76	0.54–1.07	0.80	0.57–1.12
Civil status (married/cohabiting)		–	.	–	–		**0.64**	0.44–0.93	0.69	0.47–1.01

Model 1: Unadjusted model. Model 2: Adjusted for previous depressive symptoms. Model 3: Adjusted for age, sex, children at home and civil status. Model 4: Adjusted for previous depressive symptoms, age, sex, children at home and civil status. Numbers in bold are statistically significant at *p* < 0.05

^a^If data were missing in wave two, data from the earliest available wave was used

Additional analyses were carried out using only group five as reference group (i.e. not merged with group seven). The findings remained the same, although the ORs became larger and CI’s wider. In addition, we conducted a sensitivity analysis to investigate if the results differed when including the 777 individuals with symptoms of major depression in any of the first four waves (2006–2012), increasing the sample size to 8726 (shown in Online Resource 5). Overall, OR’s were largest for the high strain and active trajectory groups across all models and the associations remained statistically significant in model 2, which indicated that our results were robust.

## Discussion

The results suggested little change in the levels of job demands and job control among Swedish employees across a 6-year period. This could potentially be explained by the fact that only 20% changed job at least once between consecutive waves. If people remain in the same job, perceived demand and control levels might not change substantially (de Lange et al. 2002). Furthermore, the results suggested that repeatedly being in a “high strain” (high demands and low control) or “active” job (high demands and high control) may have negative effects in terms of risk for subsequent symptoms of major depression. Even though some of the estimates (ORs) were no longer significant when adding covariates in model 3 and 4, there was a tendency towards higher risk for depression among those in the high strain and active trajectories. The result regarding high strain, was generally in line with previous research of the Demand-Control Model (Bonde 2008; Netterstrom et al. 2008; Nieuwenhuijsen et al. 2010; Stansfeld and Candy 2006; Theorell et al. 2015). However, it was somewhat surprising that the active group had significantly higher odds of depression in model 1 and 3. One explanation could be that the active group had a high level of demands, while the level of control was only slightly above the median. Possibly, the level of control was not high enough to mitigate the negative effects of high demands. Also, univariate trajectories of demands predicted subsequent depression to a higher extent than control trajectories, indicating that demands may be a stronger predictor of depression in this study (data not shown).

In the selected models for demands, control and their combination, the cubic, quadratic, and linear terms were often significant for all groups in addition to the intercept. However, with a few exceptions, these estimates were very small and thus of little practical significance. Because most trajectories had relatively stable levels, we were unable to examine the influence of major changes in the levels of demands and control over time. Nevertheless, the trajectories allowed us to examine repeated exposure to “high strain”, “passive”, and “active” jobs across 6 years in relation to subsequent symptoms of major depression. Another study measured job strain at three time points and found a twofold risk of subsequent depression for those exposed to repeated job strain at two or three time points compared to one (Stansfeld et al. 2012). In contrast, other scholars did not find that the number of “high strain” occurrences was related to depression (Burns et al. 2016). However, these studies did not use trajectory modelling and hence do not serve as an ideal point of comparison.

To our knowledge, only a handful of studies have examined job demand and control trajectories, and none have investigated joint demand and control trajectories in relation to depression. Nevertheless, our results are partly in line with a study, which found that average work control (trajectories) did not increase or decrease significantly across four waves (Wickrama et al. 2005). They also found that change in control influenced depression levels 7 years later.

Regarding the distribution of depression in relation to age, we found, in line with previous findings (Ferrari et al. 2013) that the youngest age group (< 35 years) had the highest proportion of major depression. Somewhat surprisingly, it was lowest in the eldest group, but this could be because individuals in this group were relatively young, most around 50–60 years old, and possibly also due to a healthy worker effect since SLOSH participants are recruited from the Swedish Work Environment Survey. Socio-economic position was not statistically associated with depression. Although a large body of evidence suggests an SES gradient, there are studies such as ours that have not been able to confirm this gradient. Incidence studies generally tend to observe a lower SES gradient (Lorant et al. 2003) and SES differentials may vary depending on setting and measurement of depression, which could explain our finding.

### Strengths and limitations

A major strength of this study is the longitudinal nature. Contrary to many previous studies with limited measurement points, we included four measurements across 6 years. By doing so, we could examine the dynamics of exposure to job demands and control over a relatively long time period, and how this was related to depression. Another strength is the large sample originating from an approximately representative sample of the Swedish working population; thereby increasing generalizability.

Despite these strengths, one limitation concerns the self-reported nature of the data. More objective measures of job demands and control, as well as clinical assessments of depression, would have been preferable and an avenue for future research. However, the entire Symptom Checklist (SCL) depression scale has been found excellent in detecting DSM-IV depressive disorder (Lundin et al. 2015). Moreover, unmeasured factors such as personality traits could possibly explain some of the relatively similar levels of demands and control, as well as the risk of depression. However, we separated our independent and dependent variables in time; thereby reducing the risk of common method bias. In addition, prediction of incident depression by a trajectory, in which observations of demands and control are modelled over time, is less likely to result from reverse causality than analyses based on measures of the exposure which do not take the development over time into account. Furthermore, we excluded individuals with symptoms of major depression in the first four waves to reduce the risk of reverse causality. However, because these individuals had higher demands and lower control, this could have led to underestimations of the association with depression. In addition, we controlled for previous depressive symptom level, as well as conducted a sensitivity analysis. However, controlling for previous symptoms may underestimate the true risks for subsequent depression in model 2 and 4 because the relatively stable exposure levels might have already influenced the risk of depression during or even before the first four waves. Therefore, one can suspect that the estimates in model 2 and 4 may be underestimated. Even though our sample originated from an approximately representative sample, another potential limitation is that the SLOSH participants who responded to the questionnaires are individuals who are more often highly educated, women, married, born in Sweden or in the Nordic countries and have high income, which limits the generalisability.

Finally, it should be noted that GBTM creates average patterns from which individual observed curves may deviate (Tu et al. 2013). With GBTM, there is also a possibility that additional unexpected, yet meaningful, trajectories exist and obtaining the optimal number of classes can be difficult (Twisk 2014). However, given our methodological rigour, we believe that our uncovered trajectories capture the most frequent patterns.

In conclusion, this study indicates that for a majority of Swedish workers the levels of job demands and job control, both separately and in combination were relatively unchanged over a time period of 6 years. Long-term exposure to a trajectory characterised by a high strain or active job may be associated with an increased risk for subsequent depression.

## Electronic supplementary material

Below is the link to the electronic supplementary material.

**Online resource Fig. 1** Flowchart of participants in the SLOSH study between 2006 and 2014, according to inclusion and exclusion criteria of this study (PDF 1057 kb)

Supplementary material 2 (PDF 63 kb)

**Online resource Fig. 3** Trajectory model of job demands in the SLOSH study. Mean level of job demands across six years according to trajectory group, and the proportion of individuals in each group (EPS 2175 kb)

**Online resource Fig. 4** Trajectory model of job control in the SLOSH study. Mean level of job control across six years according to trajectory group, and the proportion of individuals in each group (EPS 2158 kb)

Supplementary material 5 (PDF 60 kb)
