# Supplementary figures and images for: Trajectories of job demands and control: risk for subsequent symptoms of major depression in the nationally representative Swedish Longitudinal Occupational Survey of Health (SLOSH)
Source: Int Arch Occup Environ Health. 2017 Nov 11;91(3):263–72. doi: 10.1007/s00420-017-1277-0 (PMC5845058; doi:10.1007/s00420-017-1277-0)

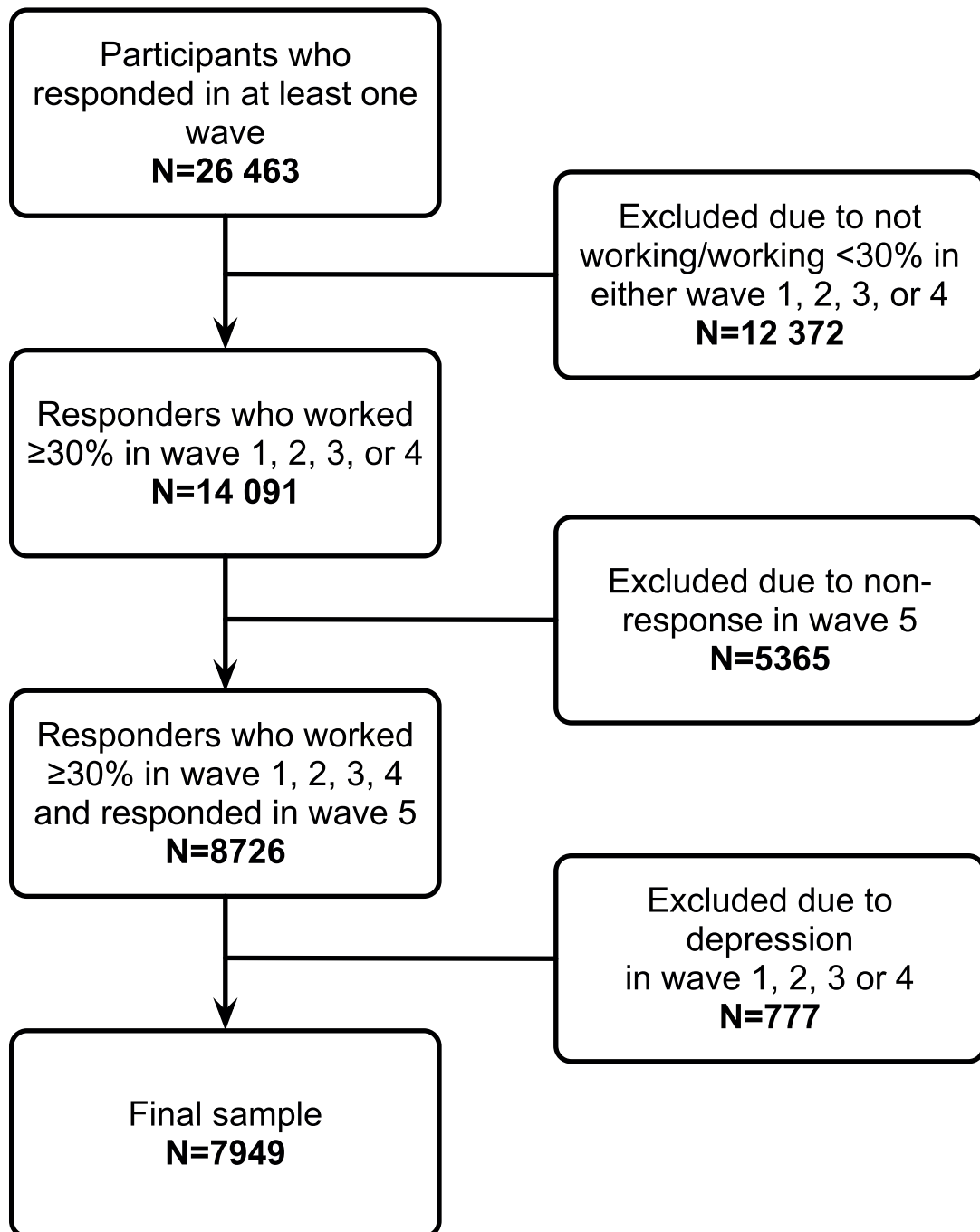

Supplement: Supplementary file 1 — Online resource Fig. 1 Flowchart of participants in the SLOSH study between 2006 and 2014, according to inclusion and exclusion criteria of this study (PDF 1057 kb) [file 420_2017_1277_MOESM1_ESM.pdf]
