# Supplementary material for: Trajectories of job demands and control: risk for subsequent symptoms of major depression in the nationally representative Swedish Longitudinal Occupational Survey of Health (SLOSH)
Source: Int Arch Occup Environ Health. 2017 Nov 11;91(3):263–72. doi: 10.1007/s00420-017-1277-0 (PMC5845058; doi:10.1007/s00420-017-1277-0)
Supplement: Supplementary file 2 — Supplementary material 2 (PDF 63 kb) [file 420_2017_1277_MOESM2_ESM.pdf]

Online resource 2 Polynomial order, fit indices and estimate of the log Bayes factor for the univariate and joint trajectory models of job demands and control

**Models**	**Demand models**			**Control models**			**Joint models**			
	Polynomial order	BIC (N=7880)	2(ΔBIC)	Polynomial order	BIC (N=7880)	2(ΔBIC)	Polynomial order	Polynomial order	BIC (N=7880)	2(ΔBIC)
One trajectory	3	-15905.65	.	2	-12173.89	-	Demands	Control		
Two trajectories	3-3	-14073.28	3664.74	3-2	-9406.43	5534.92	.	.	.	.
Three trajectories	3-3-2	-13486.69	1173.18	3-3-3	-8341.84	2129.18	.	.	.	.
Four trajectories	3-3-3-1	-13321.43	330.52	3-3-3-3	-7956.03	771.62	3-3-3-1	3-3-3-3	-23311.14	.
Five trajectories	3-3-3-1-2	-13295.50	51.86	3-3-3-2-3	-7827.31	257.44	3-3-3-1-2	3-3-3-2-3	-22660.91	1300.46
Six trajectories	**3-3-3-3-1-2**	**-13289.41**	**12.18**	**2-3-3-3-3-1**	**-7768.96**	**116.70**	3-3-3-3-1-2	2-3-3-3-3-1	-22346.11	629.6
Seven trajectories	3-1-3-3-3-1-1	-13252.31	74.20	2-3-3-3-3-2-1	-7754.48	28.96	**3-1-3-3-3-1-1**	**2-3-3-3-3-2-1**	**-21964.85**	**762.52**
Eight trajectories	3-1-3-2-2-1-1-1	-13258.13	-11.64	.	.	.	3-1-3-2-2-1-1-1	1-3-2-3-1-1-1-1	-21852.08	225.54

Polynomial order: 1=linear, 2=quadratic, 3=cubic, BIC=Bayesian information criterion
