# Supplementary material for: Trajectories of job demands and control: risk for subsequent symptoms of major depression in the nationally representative Swedish Longitudinal Occupational Survey of Health (SLOSH)
Source: Int Arch Occup Environ Health. 2017 Nov 11;91(3):263–72. doi: 10.1007/s00420-017-1277-0 (PMC5845058; doi:10.1007/s00420-017-1277-0)
Supplement: Supplementary file 5 — Supplementary material 5 (PDF 60 kb) [file 420_2017_1277_MOESM5_ESM.pdf]

Online resource 5 Results from logistic regression analysis predicting major depression in wave five presented as Odds ratios (OR) and 95% confidence intervals (CI). Sensitivity analysis, including individuals with major depression in wave 1 to 4 (2006-2012).

**Joint trajectory groups**	Depression cases n (%)	Modell 1		Modell 2		Modell 3		Modell 4	
		OR	CI	OR	CI	OR	CI	OR	CI
Low strain (5 & 7) reference	49 (2.0)								
Passive (1)	16 (5.2)	**2.61**	1.47-4.65	1.58	0.86-2.90	**2.32**	1.30-4.17	1.49	0.81-2.77
Passive (2)	27 (2.4)	1.18	0.73-1.89	1.09	0.66-1.77	1.12	0.69-1.80	1.06	0.64-1.74
High strain (3)	61 (7.8)	**4.05**	2.76-5.96	**1.94**	1.29-2.92	**3.54**	2.39-5.24	**1.86**	1.23-2.82
Hybrid (4)	89 (3.2)	**1.58**	1.11-2.26	1.23	0.85-1.78	**1.51**	1.06-2.16	1.23	0.84-1.78
Active (6)	69 (5.8)	**2.93**	2.02-4.25	**1.61**	1.09-2.38	**2.65**	1.81-3.86	**1.57**	1.05-2.33
**Covariates** [a]									
Previous depressive symptoms		.	.	**1.20**	1.18-1.22	.	.	**1.19**	1.17-1.21
Age (continuous)		.	.	.	.	**0.97**	0.96-0.98	**0.97**	0.97-0.99
Women		.	.	.	.	**1.70**	1.32-2.19	**1.39**	1.07-1.81
No children at home		.	.	.	.	0.71	0.55-0.91	0.79	0.61-1.02
Civil status (married/cohabiting)		.	.	.	.	**0.61**	0.47-0.80	0.77	0.58-1.02

Model 1: Unadjusted model. Model 2: Adjusted for previous depressive symptoms. Model 3: Adjusted for age, sex, children at home and civil status. Model 4: Adjusted for previous depressive symptoms, age, sex, children at home and civil status. Numbers in bold are statistically significant at $p<0.05$. [a] If data were missing in wave two, data from the earliest available wave was used.
